# Bioinformatics Analysis of GFAP as a Potential Key Regulator in Different Immune Phenotypes of Prostate Cancer

**DOI:** 10.1155/2021/1466255

**Published:** 2021-06-17

**Authors:** Wencheng Yao, Xiang Li, Zhankui Jia, Chaohui Gu, Zhibo Jin, Jun Wang, Bo Yuan, Jinjian Yang

**Affiliations:** ^1^Department of Urology, The First Affiliated Hospital of Zhengzhou University, Zhengzhou, Henan, China; ^2^Henan Institute of Urology, The First Affiliated Hospital of Zhengzhou University, Zhengzhou, Henan, China

## Abstract

Tumor immune escape plays an essential role in both cancer progression and immunotherapy responses. For prostate cancer (PC), however, the molecular mechanisms that drive its different immune phenotypes have yet to be fully elucidated. Patient gene expression data were analyzed from The Cancer Genome Atlas-prostate adenocarcinoma (TCGA-PRAD) and the International Cancer Genome Consortium (ICGC) databases. We used a Cell-type Identification by Estimating Relative Subsets of RNA Transcripts (CIBERSORT) analysis and an unsupervised clustering analysis to identify patient subgroups with distinct immune phenotypes. These distinct phenotypes were then explored for associations for differentially expressed genes (DEGs) and both epigenetic and genetic landscapes. Finally, we used a protein-protein interaction analysis to identify key hub genes. We identified two patient subgroups with independent immune phenotypes associated with the expression of Programmed death-ligand 1 (PD-L1). Patient samples in Cluster 1 (*C*1) had higher scores for immune-cell subsets compared to Cluster 2 (*C*2), and *C*2 samples had higher specific somatic mutations, MHC mutations, and genomic copy number variations compared to *C*1. We also found additional cluster phenotype differences for DNA methylation, microRNA (miRNA) expression, and long noncoding RNA (lncRNA) expression. Furthermore, we established a 4-gene model to distinguish between clusters by integrating analyses for DEGs, lncRNAs, miRNAs, and methylation. Notably, we found that glial fibrillary acidic protein (GFAP) might serve as a key hub gene within the genetic and epigenetic regulatory networks. These results improve our understanding of the molecular mechanisms underlying tumor immune phenotypes that are associated with tumor immune escape. In addition, GFAP may be a potential biomarker for both PC diagnosis and prognosis.

## 1. Introduction

Prostate cancer (PC) is the most frequently diagnosed type of cancer and ranks second among the leading causes of cancer-related deaths in men [1]. Although early detection has improved significantly, and the 5-year survival rate for early-stage cancer is excellent, the rate of false-positives is high for the prostate-specific antigen (PSA) screening test, and prostate metastatic disease is associated with both poor outcomes and deceptively low serum PSA levels that can lead to false-negative results [2]. Thus, it is critical to identify novel prognostic biomarkers and therapeutic strategies for improving overall survival (OS) for patients with PC.

Several studies have demonstrated that the tumor microenvironment has both tumor-antagonizing and tumor-promoting roles. Tumor-antagonizing immune cells mainly consist of M1-polarized macrophages, CD8^+^ cytotoxic T cells, effector CD4^+^T cells, natural killer (NK) cells, dendritic cells (DCs), and N1-polarized neutrophils which are able to either present tumor cells, kill them directly, or secrete cytokines that interact with malignant cells [3, 4]. Tumor-promoting immune cells mainly include regulatory T cells (Tregs) and myeloid-derived suppressor cells (MDSCs) [3]. Notably, cancer cells can eventually evade immune surveillance through a variety of mechanisms and resist the cytotoxic effects of immune cells. As a new hallmark of cancer, this ability of immune escape provides new opportunities for cancer treatment strategies. Recently, four groups of microenvironment immune types have been categorized using genomic analysis of 14 types of solid cancers. However, considering their complexity and the dynamic nature of immune cells, it is evident that a more in-depth evaluation of the associations between immune cells and PC is needed.

The programmed cell death-1 receptor (PD-1) is a representative immune checkpoint inhibitor expressed in activated T cells, B cells, and macrophages. Programmed death-ligand 1 (PD-L1), a ligand of PD-1, is often found on both tumor cells and antigen-presenting cells and provides potent inhibitory interactions within the tumor microenvironment [5]. The major function of PD-L1 limits tumor cells from evading immunity, but unfortunately, this has become a main mechanism for immune resistance in the immune microenvironment. In addition, the expression of PD-L1 in tumors is often regarded as a negative prognostic factor, but it is clearly a positive factor for PD-1/PD-L1 treatments [6].

In this PC study, we established two patient-sample clusters with different immunophenotypes using an integrative multiomic approach to better understand the molecular mechanisms of tumor immune escape and found the key regulatory node within the genetic and epigenetic regulatory networks. Furthermore, this key regulatory node may be a novel tumor immune escape predictor and a promising target for PC therapy.

## 2. Materials and Methods

### 2.1. Patients and Gene Expression Profiles

Clinical and RNA-expression patient data in pan-cancer were collected from the prostate adenocarcinoma dataset of The Cancer Genome Atlas (TCGA-PRAD; https://portal.gdc.cancer.gov/) and TCGA-PRAD serves as training datasets. The cohorts used for validation studies were downloaded from the International Cancer Genome Consortium (ICGC; https://dcc.icgc.org/). All patient data that were used in the present study had complete clinical information, including age, sex, grade, TNM stage, and overall survival time. The PRAD protein-expression data were obtained from https://http://www.tcpaportal.org/tcpa. Functional lncRNA and microRNA data were downloaded from the TCGA data portal (https://portal.gdc.cancer.gov/) [7].

### 2.2. Cell-Type Identification by Estimating Relative Subsets of RNA Transcripts (CIBERSORT) Analyses

The CIBERSORT computational approach is commonly used to predict the infiltration of 22 types of immune cells from the gene expression profiles of complex tissues (http://cibersort.stanford.edu) [8]. The 22 types of immunes cells include 7 types of T cells, 3 types of macrophages, naïve B cells, memory B cells, activated NK cells, plasma cells, monocytes, resting natural killer (NK) cells, resting mast cells, activated mast cells, resting dendritic, cells, activated dendritic cells, neutrophils, and eosinophils.

In the present study, CIBERSORT is used to calculate the absolute immune-cell fraction in the PC samples (model = absolute, permutation = 1,000, disable quantile normalization for RNA-Seq data as recommended) using the method of estimating the relative subset of RNA transcript for cell type identification.

### 2.3. Unsupervised Hierarchical Cluster Analysis

Immune-cell expression values were clustered using an unsupervised hierarchical clustering method (Euclidean distance and ward linkage) provided by the dendextend R software package [9]. The ClustVis tool (http://biit.cs.ut.ee/clustvis/) was used to process and modify these data for the final plotting of the principal component analysis and the heat map [10].

#### 2.3.1. Comparing Somatic Mutation Frequencies and Somatic Copy Number Variants (CNVs)

To assess the relative frequency of somatic mutation between the different immunophenotypes, the somatic mutation sequencing data from TCGA-PRAD clinical samples were acquired from cBioPortal (http://www.cbioportal.org/) [11] and were analyzed using the Complex Heatmap R software package [12]. Total gene mutations frequencies ≥ 5% for DNA damage responses and repair factors were identified. In addition, the characteristic molecular profiles of the cohorts were determined.

Level 3 CNV data from TCGA were retrieved from the firebrowse data portal (http://firebrowse.org/) using a threshold of 0.2 for segmented mean amplification values and −0.2 for deletion values. Fisher's exact test was used for determining statistical significance (http://convaq.combio.sdu.dk/), using the average number of deletions and duplications per sample as background data, and the number of samples with deletions and amplifications for the loci [13]. The CNV summary diagram was generated by IGV-2.2.19, and the Circos figure was analyzed using the RCircos R software package.

### 2.4. The Single-Sample Gene Set Enrichment Analysis (ssGSEA)

As described previously, ssGSEA was used to score each sample based on the list of screened genes in the TCGA-PRAD cohort. To determine any correlations between the immune phenotypes and the screened genes and to verify grouping accuracy, we use the ssGSEA algorithm to calculate the ssGSEA enrichment score of immune genes in the ICGC-PRAD-CA cohort based on the published immune-related gene set genes (doi:10.1172/JCI91190).

### 2.5. Target-Gene Predictions for MicroRNAs (miRNAs) and Long Noncoding RNAs (lncRNAs)

To study miRNA functionality, the target mRNAs for identified miRNAs were predicted using DIANA (http://diana.imis.athena-innovation.gr/DianaTools/index.php). The screening threshold used was a miTG score of ≥0.95. Target-gene predictions for identified lncRNAs were made using LncRNA2Target software (http://123.59.132.21/lncrna2target/download.jsp).

### 2.6. DNA Methylation Analysis

The DNA methylation analyses were performed using Illumina 450K chip-based information from the TCGA-PRAD database (http://gdc.xenahubs.net/download/TCGA-PRAD.methylation450.tsv.gz/). The methylation data were standardized using the watermelon package in R software, and the probes for differential methylations were detected using the minfi package in R software.

### 2.7. Analysis of Protein-Protein Interactions (PPIs)

The PPI network was analyzed using the STRING Consortium online database (https://string-db.org/). In Cytoscape (version 3.6.1) software, the PPI network was analyzed using Cytoscape plugin-MCODE (for clustering of the PPI network) and cytoHubba (for identifying hub proteins), as described previously [14].

### 2.8. Statistical Analyses

All statistical analyses were performed using SPSS 25 (IBM, Chicago, IL, USA) and GraphPad Prism 7.0 (GraphPad Software, La Jolla, CA, USA). The versions of R software used were more recent than v.3.5.1. For the correlation analyses, Student's *t*-tests were used. A univariate analysis was performed to analyze possible prognostic factors. All statistical results with *p* < 0.05 were considered statistically significant.

## 3. Results

### 3.1. Identification of the Immune-Cell Subsets Related to PD-L1 Expression in PC Samples

**Figure 1:**
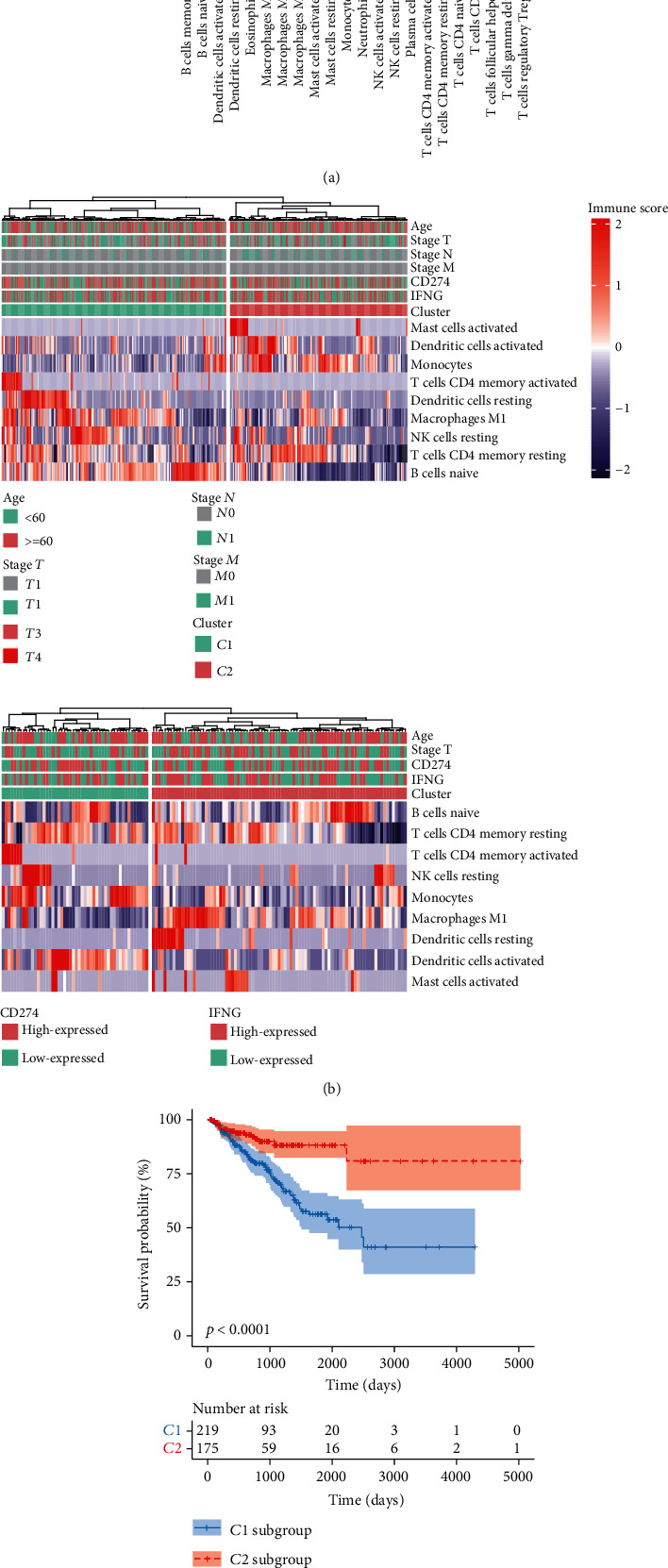
The immune-related subtypes for prostate cancer. (a) Correlations between the expression of PD-L1 and immune-cell infiltration ratios in TCGA-PRAD and ICGC-PRAD cohorts. (b) The distribution of immune-related subtypes and associated clinical characteristics of TCGA (top) and ICGC (bottom) cohorts. (c) Kaplan-Meier analysis of groups *C*1 and *C*2 in TGCA-PRAD cohort.

To evaluate the infiltrations of different immune-cell subtypes, a training set (TCGA-PRAD, *n* = 519) and a validation cohort (ICGC-PRAD-CA, *n* = 144) were used for the CIBERSORT analysis. Overall, nine immune-cell subtypes (activated mast cells, monocytes, M1 macrophages, activated dendritic cells, naive B cells, activated memory T cells-CD4, resting dendritic cells, resting NK cells, and resting memory T cells-CD4) were identified as being positively correlated with PD-L1 transcript levels (Figure 1(a)). These nine cell types were then further characterized.

### 3.2. Classification of PC Samples into Subgroups Based on Immune-Cell Subsets

To better describe the relationship between tumor-infiltrating immune cells and tumor-cell immune escape, we classified the PC sample data into two major clusters (*C*1 and *C*2) based on the above nine immune-cell subsets (Figure 1(b)). Patient samples in group *C*1 had higher scores for selected immune-cell subsets compared to *C*2 samples (Figure 1(b)), so *C*1 patient samples were defined as having a high cell-cytotoxic immune phenotype, and *C*2 patient samples were defined as having a low cell-cytotoxic immune phenotype. The same findings were also verified using the ICGC-PRAD-CA validation cohort. A Kaplan-Meier analysis demonstrated that *C*1 patients also had shorter overall survive times than *C*2 patients according to TCGA-PRAD dataset (Figure 1(c)).

### 3.3. Differences in MHC Class I Gene Expression Related to Immune Phenotypes

MHC class I (MHC-I) tumor antigens expressed on tumor-cell surfaces determine the capacity of cytotoxic T lymphocytes (CTLs) to recognize and eliminate tumor cells. The lack of MHC-I antigens on CTLs is an important mechanism leading to tumor-cell immune evasion. The expression of MHC-I antigens on cancer cells also affects tumor responses to immunotherapy.

**Figure 2:**
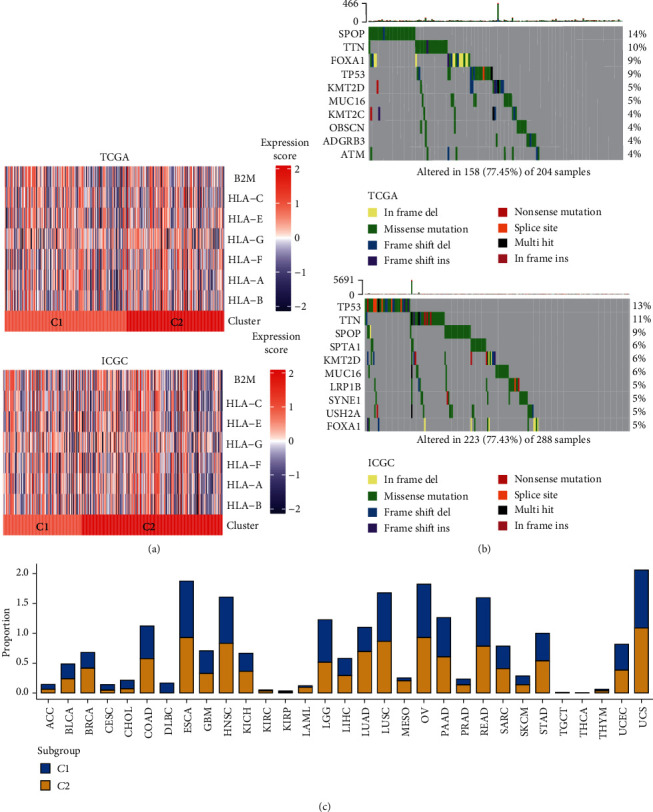
Differences in MHC class I gene expression and somatic mutations between the two immune phenotypes. (a) The transcription levels for B2M and for human leukocyte antigen (HLA) genes encoding MHC class I protein in TCGA-PRAD (top) and ICGC-PRAD cohorts (bottom). (b) The top-10 mutated genes in groups *C*1 (top) and *C*2 (bottom) in thePRAD database. (c) In TCGA-PRAD cohort, the mutation frequency of TP53 in group *C*2 was relatively higher than in group *C*1 for most types of cancer.

We therefore evaluated the transcript levels of *β*2-microglobulin (B2M) and human leukocyte antigen (HLA) genes encoding MHC class I protein in the independent TCGA-PRAD validation cohort. The results showed that *C*2 expressions were lower than *C*1 expressions (Figure 2(a)), indicating impaired antigen presentation for *C*2 tumor cells and an escape mechanism for *C*2 immune surveillance. The same results were obtained using the independent ICGC-PRAD-CA validation cohort (Figure 2(a)).

### 3.4. Somatic Mutation Differences between Immune Phenotypes

To understand changes at the gene level, we analyzed the number and quality of somatic mutations in the *C*1 and *C*2 subgroups of the TCGA-PRAD cohort. We found that the TP53 mutation frequency in group *C*2 was higher than that in group *C*1, and the 10 most-mutated genes are shown in Figure 2(b). Similar to the TCGA-PRAD results, the mutation frequency of TP53 in *C*2 was relatively high in most types of TCGA cohort cancers (Figure 2(c)).

### 3.5. Differences in Genomic CNVs between Immune Phenotypes

**Figure 3:**
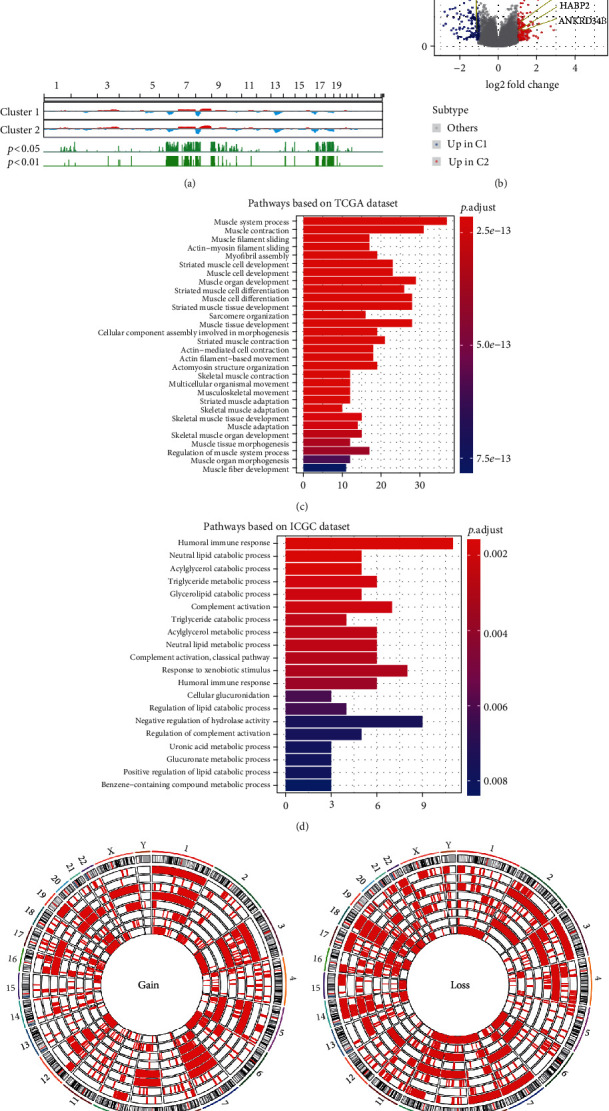
Differences in genomic copy number variations (CNVs) related to the two immune phenotypes. (a) CNV statistics between groups *C*1 and *C*2 in TCGA-PRAD cohort. (b) The distribution of differentially expressed genes (DEGs) between groups *C*1 and *C*2. (c) GO enrichment analysis of DEGs in group *C*1. (d) GO enrichment analysis of DEGs in group *C*2. (e and f) CNVs compared among six other tumor types in TCGA cohorts. Cohorts from outside to inside diameters: PRAD, GBM, KIRP, LGG, PAAD, SARC, and TGCT.

Studies have found that genomic CNVs are closely associated with immune system evasion [15, 16]. Analyses of changes in the genomes of TCGA-PRAD cohort showed that group *C*2 had significant amplifications (chromosomes 1q, 4q, 14q, and 17q) and significant deletions (chromosomes 7q, 8p, 14q, and 19q) in several hotspot regions compared to group *C*1 (Figure 3(a)). To assess whether CNVs affected gene expression, both *C*1 and *C*2 were screened for DEGs, and the results showed that 134 genes were upregulated in group *C*1 and 135 genes were upregulated in group *C*2 (Figure 3(b)). GO enrichment analysis determined that the upregulated *C*1 genes were involved in immune-cell adhesion and movement, functions related to the activation of immune cells (Figure 3(c)). Upregulated *C*2 genes were involved in functions related to keratinization, development, and differentiation (Figure 3(d)). Similar to the PRAD dataset results, several TCGA cohorts (glioblastoma multiforme (GBM), kidney renal papillary cell carcinoma (KIRP), brain lower-grade glioma (LGG), liver hepatocellular carcinoma (LIHC), pancreatic adenocarcinoma (PAAD), sarcoma (SARC), testicular germ cell tumor (TGCT), and prostate adenocarcinoma (PRAD)) shared significantly enriched deletions in chromosomes 6p, 10p, and 18p, and increased copy numbers of chromosome 1q in group *C*2 compared to group *C*1 (Figures 3(e) and 3(f)). Collectively, the results indicate that group *C*2 cancer cells adapt to the presence of tumor-infiltrating lymphocytes by acquiring specific somatic mutations, MHC mutations, and genomic CNVs, and group *C*1 escapes immune surveillance by changing the activation and proliferation of immune cells in the immune microenvironment.

### 3.6. Differences in DNA Methylation and miRNA Expression between Immune Phenotypes

**Figure 4:**
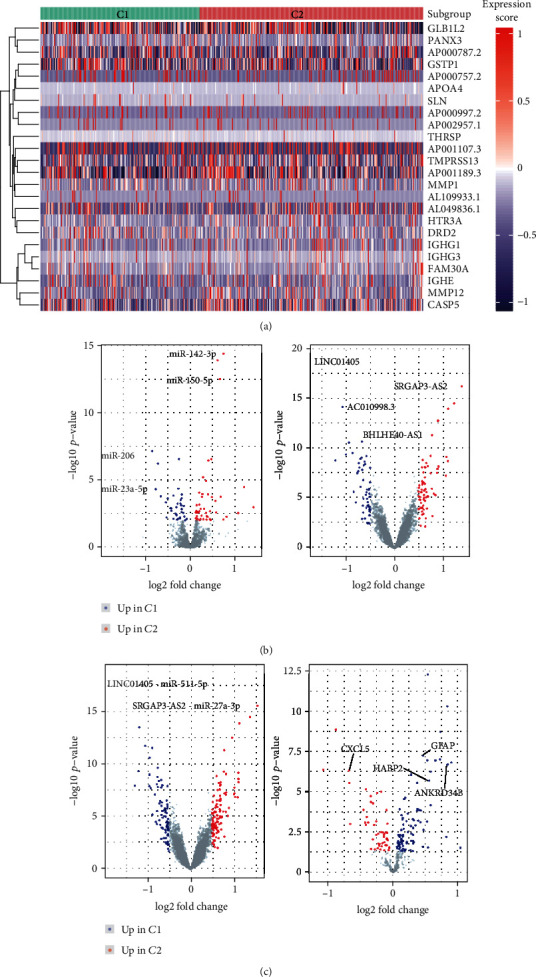
The relationship between miRNAs, lncRNAs, and differentially expressed genes (DEGs) between the two subtypes. (a) Analysis of differentially-methylated gene expression between groups *C*1 and *C*2. (b) Differentially expressed miRNAs (top left) and differentially expressed lncRNAs (top right) between the two subgroups. (c) Differentially expressed lncRNA-miRNA links (bottom left) and DEGs (bottom right) between the two subgroups.

Aberrant DNA methylation is associated with both cellular identity and tumor immune surveillance [17]. To explore how DNA methylation affected tumor immunophenotype, we analyzed genome-wide methylation data in the TCGA-PRAD cohort. In total, we found 43 differentially methylated regions between groups *C*1 and *C*2 (FDR < 0.05), and a total of 33 gene sequences were represented by these regions. The regions with higher beta values for group *C*1 compared to group *C*2 had relatively low gene expression levels (Figure 4(a)).

Next, 83 differentially expressed miRNAs (significance = −1 < fold − change (FC) or FC > 1, false-discovery rate (FDR) < 0.01) were assessed between groups *C*1 and *C*2 based on TCGA-PRAD cohort (Figure 4(b)). Considering that identifying the target genes for these miRNAs would be useful for understanding miRNA regulatory functions, we identified 5185 target genes and 10437 gene links. Finally, we also identified 166 links for DEGs (−1.41 < FC or FC > 1.41, FDR < 0.01), of which 101 were upregulated in group *C*1 and 65 were upregulated in group *C*2 (Figure 4(b)).

### 3.7. Differences in lncRNA Expression between Immune Phenotypes

lncRNAs compete with miRNAs by occupying shared binding sequences, thereby sequestering miRNAs and changing the expression of their downstream target genes. Interaction networks between lncRNAs, miRNAs, and mRNAs have been documented for a variety of biological processes in many diseases [18]. Here, we found 118 differentially expressed lncRNAs (significance = −0.5 < log2FC > 0.5, FDR < 0.05) between groups *C*1 and *C*2 using TCGA-PRAD cohort, with 53 of them highly expressed in group *C*1, and 65 highly expressed in group *C*2 (Figure 4(c)). In addition, our results revealed 2752820 lncRNA-miRNA links, including 2588 miRNAs and 14666 lncRNAs from the miRcode database. By combining the differentially expressed lncRNA analysis with the lncRNA-miRNA links information from the miRcode database, we documented 46 differentially expressed lncRNAs that interacted with miRNAs and 746 lncRNA-miRNA links. We next used the LncRNA2Target database to predict the target genes for these lncRNAs. A total of 138 DEGs were found to interact with these lncRNAs. Among them, 88 targeted DEGs were upregulated in group *C*1, and 50 targeted DEGs were upregulated ingroup *C*2 (Figure 4(c)).

### 3.8. Glial Fibrillary Acidic Protein (GFAP) as a Key Node within the Genetic and Epigenetic Regulatory Networks

**Figure 5:**
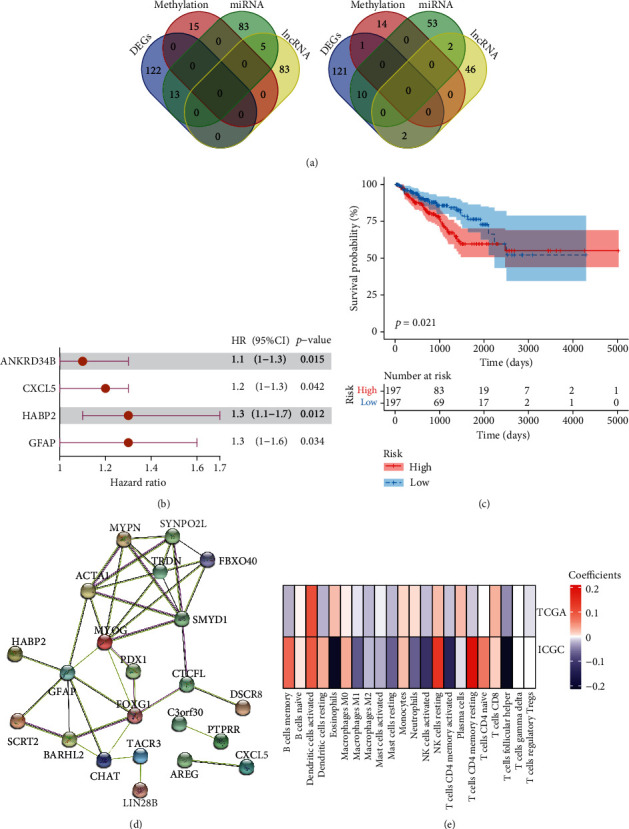
Identification of a key node within the genetic and epigenetic regulatory networks. (a) The integration of DEG, lncRNA, miRNA, and methylation analyses to determine upregulated differentially expressed genes in groups *C*1 and *C*2. (b) The four genes positively associated with prognosis were confirmed using a random forest blot in TCGA-PRAD cohort. (c) A Kaplan-Meier analysis of the 4-gene model using TGCA-PRAD cohort. (d) GFAP was identified as a key hub gene within the network using a protein-protein interaction analysis of the STRING Consortium database. (e) Spearman's correlation analysis was performed to analyze correlations between GFAP transcription levels and immune-cell subsets for TCGA-PRAD cohort.

Cancer is the result of complicated interactions between genetic and epigenetic variations [19], and we considered that these two tumor immunophenotypes might be driven by both genetic and epigenetic regulators. We identified 13 DEGs that were increased in group *C*1 and 13 DEGs were increased in group *C*2 by integrating the analyses of DEGs, lncRNAs, miRNAs, and methylation (Figure 5(a)). We used a single-factor Cox regression for these 26 genes and identified four genes that were significantly associated with PC prognosis. Three of these four genes were highly expressed in group *C*1, and one of the four was highly expressed in group *C*2. As expected, the ssGSEA scores based on these genes were confirmed for both the *C*1 and *C*2 groups using TCGA-PRAD cohort and the independent validation ICGC-PRAD cohort (Supplementary Figure 1). In addition, the ssGSEA scores were further confirmed for both the *C*1 and *C*2 groups for other tumor typesin TCGA cohort (Supplementary Figure 2).

These four identified genes were also confirmed using a random forest blot from TCGA-PRAD cohort data (Figure 5(b)). After calculating the risk scores for the four gene sets using a multivariate Cox regression analysis, they were used for a prognosis analysis of data from the TCGA-PRAD cohort. These four genes were found to have excellent predictive ability for PC prognoses (*p* < 0.05, Figure 5(c)).

Importantly, we identified GFAP as a key hub gene within the network using PPIs from the STRING Consortium database (Figure 5(d)), and we used Spearman's correlation analysis to assess any correlations between GFAP transcription levels and immune-cell subsets in the TCGA-PRAD cohort. We found that GFAP expression was positively correlated with T cell CD4 memory resting and NK cells resting, while GFAP expression was natively correlated with T cells follicular helper, eosinophils, NK cells activated, plasma cells, and macrophage M1 (Figure 5(e)). The results confirmed that high GFAP expression was positively correlated with immunosuppression.

## 4. Discussion

Tumor-associated immune cells are an important part of the tumor microenvironment. A growing number of evidences show that immune cells in tumor microenvironment play a vital role in the initiation and progression in prostate cancer [20]. The infiltrating immune cells in the prostate tumor microenvironment mainly include macrophages, neutrophils, myeloid-derived suppressor cells (MDSCs), and T regulatory cells (Tregs) [21]. In the present study, the results of CIBERSORT analyses showed that 9 immune-cell subtypes (activated mast cells, monocytes, M1 macrophages, activated dendritic cells, naive B cells, activated memory T cells-CD4, resting dendritic cells, resting NK cells, and resting memory T cells-CD4) were positively correlated with PD-L1 transcript levels. To better describe the relationship between tumor-infiltrating immune cells and tumor-cell immune escape, we classified the PC sample data into two major clusters (*C*1 and *C*2) based on the above nine immune-cell subsets.

Different immune subtypes have been identified among patients with cancer, a promising approach for understanding the effects of the immune microenvironment on tumors. For hepatocellular carcinoma, Sia et al. also identified two subgroups characterized by exhausted or adaptive immune responses to predict the therapeutic effects of PD-1, PD-L1, or transforming growth factor *β*1 inhibitor [22]. Similarly, Li et al. reported both immune-reduced and immune-enhanced subtypes with differing immune-related and clinical characteristics to provide targets for new treatments [23]. For breast cancer, Zheng et al. developed an immune phenotype classifier for predicting both immune activity within the tumor microenvironment and prognoses for patients with triple-negative breast cancer [24]. In addition, numerous investigations have been conducted using immune subtypes to predict clinical prognoses and treatment guidance for bladder cancer [25], ovarian carcinoma [26], uveal melanoma [27], lung cancer [28, 29], and head and neck cancers [30]. Here, we have identified two immune system-related PC phenotypes with differing transcription level scores among immune-cell subsets using a CIBERSORT analysis and further characterized their differences using a multiomic approach. The results revealed that identifying immune-related cancer subtypes was meaningful for both a better understanding of tumor molecular mechanisms and for clinical prognoses.

Emerging evidence suggests that both genetic and epigenetic alterations in cancer cells drive malignancy. Here, we have highlighted the genetic mutations and epigenetic aberrations driving different PC immunophenotypes. Similarly, Feng et al. described the mutational and epigenetic landscape for head and neck cancers using immune-related phenotypes [30]. For glioblastomas, tumor drug tolerance would evolve by acquiring genetic and epigenetic alterations [31]. For ovarian cancers, both genetic and epigenetic factors likely contribute to shaping the immunosuppressive tumor microenvironment and to improving the response rate of ovarian cancer to immune checkpoint therapies [32]. Similarly, for acute myeloid leukemia, genetic abnormalities and aberrant epigenetic regulators both play essential roles that affect responses to therapy and prognoses [33].

By integrating the genetic and epigenetic alterations and their effects on DEGs between these two immune subgroups, we found that GFAP was the key hub gene in this regulation network. Previous research has demonstrated that the expression of GFAP was aberrant in astrocytoma tissue compared to normal brain tissue [34–38], with GFAP levels decreasing as with astrocytoma grading increased [39–41], so GFAP levels may serve as a novel cancer diagnostic marker.

## 5. Conclusions

The present data provide further insight into the underlying molecular mechanisms for alterations to both the mutational landscape and the epigenome for these two immunophenotypes. GFAP, a key node among mechanistic pathways, may be a potential biomarker both for PC diagnosis and for predicting prognoses.

## Data Availability

Publicly available datasets were analyzed in this study.

## Supplementary Materials

Supplementary Materials Supplementary Figure 1: comparisons of ssGSEA scores from TCGA-PRAD cohort. (a) The ssGSEA scores from all screened genes between the two subgroups in the TCGA-PRAD cohort. (b) The ssGSEA scores from the two subgroups based on *C*1-upregulated genes in the TCGA-PRAD cohort. (c) The ssGSEA scores from the two subgroups based on *C*2-upregulated genes in the TCGA-PRAD cohort. Supplementary Figure 2: the ssGSEA scores based on *C*1- and *C*2-upregulated genes. (a) The ssGSEA scores for *C*1-upregulated genes between *C*1 and *C*2 subgroups were applied to total tumor entities in the TGCA cohort. (b) The ssGSEA scores based on *C*2-upregulated genes between the *C*1 and *C*2 subgroups applied to total tumor entities in the TCGA cohort.

## References

[1] Siegel R. L., Miller K. D., Jemal A. (2018). Cancer statistics, 2018. *CA: a Cancer Journal for Clinicians*.

[2] Center M. M., Jemal A., Lortet-Tieulent J. (2012). International variation in prostate cancer incidence and mortality rates. *European Urology*.

[3] Lei X., Lei Y., Li J. K. (2020). Immune cells within the tumor microenvironment: biological functions and roles in cancer immunotherapy. *Cancer Letters*.

[4] Smyth M. J., Dunn G. P., Schreiber R. D. (2006). Cancer immunosurveillance and immunoediting: the roles of immunity in suppressing tumor development and shaping tumor immunogenicity. *Advances in Immunology*.

[5] Schütz F., Stefanovic S., Mayer L., von Au A., Domschke C., Sohn C. (2017). PD-1/PD-L1 pathway in breast cancer. *Oncol Res Treat.*.

[6] Balar A. V., Weber J. S. (2017). PD-1 and PD-L1 antibodies in cancer: current status and future directions. *Cancer Immunology, Immunotherapy*.

[7] Chu A., Robertson G., Brooks D. (2016). Large-scale profiling of microRNAs for the Cancer Genome Atlas. *Nucleic Acids Research*.

[8] Newman A. M., Liu C. L., Green M. R. (2015). Robust enumeration of cell subsets from tissue expression profiles. *Nature Methods*.

[9] Galili T. (2015). Dendextend: an R package for visualizing, adjusting and comparing trees of hierarchical clustering. *Bioinformatics*.

[10] Metsalu T., Vilo J. (2015). ClustVis: a web tool for visualizing clustering of multivariate data using principal component analysis and heatmap. *Nucleic Acids Research*.

[11] Cerami E., Gao J., Dogrusoz U. (2012). The cBio cancer genomics portal: an open platform for exploring multidimensional cancer genomics data. *Cancer Discovery*.

[12] Gu Z., Eils R., Schlesner M. (2016). Complex heatmaps reveal patterns and correlations in multidimensional genomic data. *Bioinformatics*.

[13] Laddha S. V., Ganesan S., Chan C. S., White E. (2014). Mutational landscape of the essential autophagy gene BECN1 in human cancers. *Molecular Cancer Research*.

[14] Yang Y., Chen D., Liu H., Yang K. (2019). Increased expression of lncRNA CASC9 promotes tumor progression by suppressing autophagy-mediated cell apoptosis via the AKT/mTOR pathway in oral squamous cell carcinoma. *Cell Death & Disease*.

[15] Bhattacharya A., Bense R. D., Urzúa-Traslaviña C. G., de Vries E. G. E., van Vugt M., Fehrmann R. S. N. (2020). Transcriptional effects of copy number alterations in a large set of human cancers. *Nature Communications*.

[16] Grasso C. S., Giannakis M., Wells D. K. (2018). Genetic mechanisms of immune evasion in colorectal cancer. *Cancer Discovery*.

[17] Li J., Li Y., Li W. (2019). Guide positioning sequencing identifies aberrant DNA methylation patterns that alter cell identity and tumor-immune surveillance networks. *Genome Research*.

[18] Wang L., Cho K. B., Li Y., Tao G., Xie Z., Guo B. (2019). Long noncoding RNA (lncRNA)-mediated competing endogenous RNA networks provide novel potential biomarkers and therapeutic targets for colorectal cancer. *International Journal of Molecular Sciences*.

[19] Zhou S., Treloar A. E., Lupien M. (2016). Emergence of the noncoding cancer genome: a target of genetic and epigenetic alterations. *Cancer Discovery*.

[20] Hu P., Gao Y., Huang Y. (2020). Gene expression-based immune cell infiltration analyses of prostate cancer and their associations with survival outcome. *DNA and Cell Biology*.

[21] Wu S. Q., Su H., Wang Y. H., Zhao X. K. (2019). Role of tumor-associated immune cells in prostate cancer: angel or devil?. *Asian Journal of Andrology*.

[22] Sia D., Jiao Y., Martinez-Quetglas I. (2017). Identification of an immune-specific class of hepatocellular carcinoma, based on molecular features. *Gastroenterology*.

[23] Li W., Wang H., Ma Z. (2019). Multi-omics analysis of microenvironment characteristics and immune escape mechanisms of hepatocellular carcinoma. *Frontiers in Oncology*.

[24] Zheng S., Zou Y., Xie X. (2020). Development and validation of a stromal immune phenotype classifier for predicting immune activity and prognosis in triple-negative breast cancer. *International Journal of Cancer*.

[25] Song B. N., Kim S. K., Mun J. Y., Choi Y. D., Leem S. H., Chu I. S. (2019). Identification of an immunotherapy-responsive molecular subtype of bladder cancer. *eBioMedicine*.

[26] Zheng M., Hu Y., Gou R. (2020). Identification of immune-enhanced molecular subtype associated with BRCA1 mutations, immune checkpoints and clinical outcome in ovarian carcinoma. *Journal of Cellular and Molecular Medicine*.

[27] Pan H., Lu L., Cui J., Yang Y., Wang Z., Fan X. (2020). Immunological analyses reveal an immune subtype of uveal melanoma with a poor prognosis. *Aging (Albany NY)*.

[28] Qin F. L., Xu Z. Y., Yuan L. Q. (2020). Novel immune subtypes of lung adenocarcinoma identified through bioinformatic analysis. *FEBS Open Bio*.

[29] Kurebayashi Y., Emoto K., Hayashi Y. (2016). Comprehensive immune profiling of lung adenocarcinomas reveals four immunosubtypes with plasma cell subtype a negative indicator. *Cancer Immunology Research*.

[30] Feng B., Shen Y., Pastor Hostench X. (2020). Integrative analysis of multi-omics data identified EGFR and PTGS2 as key nodes in a gene regulatory network related to immune phenotypes in head and neck cancer. *Clinical Cancer Research*.

[31] Eyler C. E., Matsunaga H., Hovestadt V., Vantine S. J., van Galen P., Bernstein B. E. (2020). Single-cell lineage analysis reveals genetic and epigenetic interplay in glioblastoma drug resistance. *Genome Biology*.

[32] Cho Y., Milane L., Amiji M. M. (2019). Genetic and epigenetic strategies for advancing ovarian cancer immunotherapy. *Expert Opinion on Biological Therapy*.

[33] Bret C., Viziteu E., Kassambara A., Moreaux J. (2016). Identifying high-risk adult AML patients: epigenetic and genetic risk factors and their implications for therapy. *Expert Review of Hematology*.

[34] Chumbalkar V. C., Subhashini C., Dhople V. M. (2005). Differential protein expression in human gliomas and molecular insights. *Proteomics*.

[35] Laczko R., Szauter K. M., Jansen M. K. (2007). Active lysyl oxidase (LOX) correlates with focal adhesion kinase (FAK)/paxillin activation and migration in invasive astrocytes. *Neuropathology and Applied Neurobiology*.

[36] Sharpe M. A., Baskin D. S. (2016). Monoamine oxidase B levels are highly expressed in human gliomas and are correlated with the expression of HiF-1*α* and with transcription factors Sp1 and Sp3. *Oncotarget*.

[37] Bien-Möller S., Balz E., Herzog S. (2018). Association of glioblastoma multiforme stem cell characteristics, differentiation, and microglia marker genes with patient survival. *Stem Cells International*.

[38] Su X., Liu X., Ni L. (2016). GFAP expression is regulated by Pax 3 in brain glioma stem cells. *Oncology Reports*.

[39] van Bodegraven E. J., van Asperen J. V., Robe P. A. J., Hol E. M. (2019). Importance of GFAP isoform-specific analyses in astrocytoma. *Glia*.

[40] Tichy J., Spechtmeyer S., Mittelbronn M. (2016). Prospective evaluation of serum glial fibrillary acidic protein (GFAP) as a diagnostic marker for glioblastoma. *Journal of Neuro-Oncology*.

[41] Gállego Pérez-Larraya J., Paris S., Idbaih A. (2014). Diagnostic and prognostic value of preoperative combined GFAP, IGFBP-2, and YKL-40 plasma levels in patients with glioblastoma. *Cancer*.

