# Supplementary material for: Bioinformatics Analysis of GFAP as a Potential Key Regulator in Different Immune Phenotypes of Prostate Cancer
Source: Biomed Res Int. 2021 Jun 17;2021:1466255. doi: 10.1155/2021/1466255 (PMC8225431; doi:10.1155/2021/1466255)

**Supplementary Figures**

**Supplementary Figure 1.** Comparisons of ssGSEA scores from TCGA-PRAD cohort. A. The ssGSEA scores from all screened genes between the two subgroups in TCGA-PRAD cohort. B. The ssGSEA scores from the two subgroups based on C1-upregulated genes in TCGA-PRAD cohort. C. The ssGSEA scores from the two subgroups based on C2-upregulated genes in TCGA-PRAD cohort.

**Supplementary Figure 2.** The ssGSEA scores based on C1- and C2-upregulated genes. A. The ssGSEA scores for C1-upregulated genes between C1 and C2 subgroups applied to total tumor entities in TGCA cohort. B. The ssGSEA scores based on C2-upregulated genes between the C1 and C2 subgroups applied to total tumor entities in TCGA cohort.

**Supplementary Figure 1**


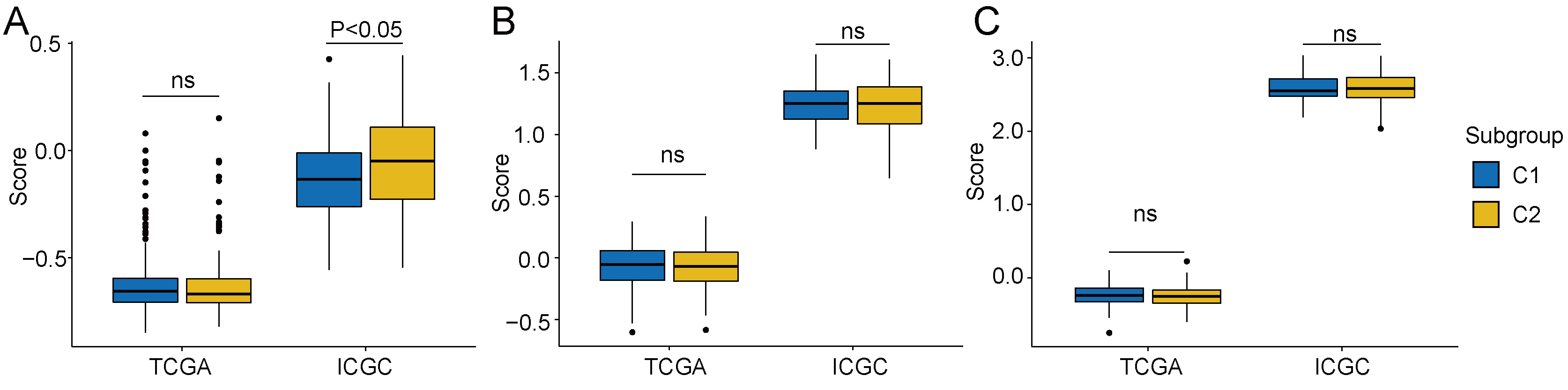


**Supplementary Figure 2**


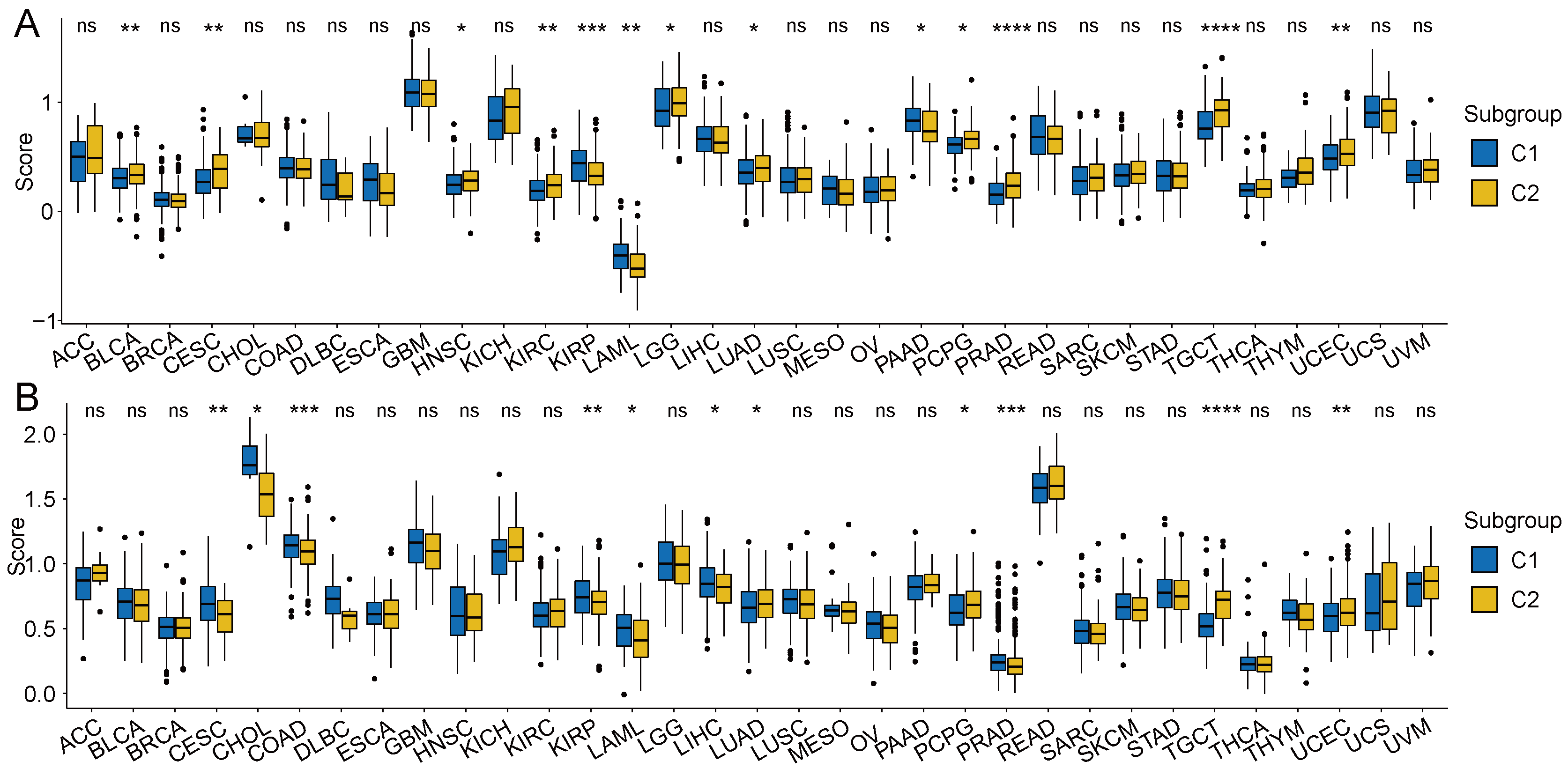

Supplement: Supplementary Materials — Supplementary Figure 1: comparisons of ssGSEA scores from TCGA-PRAD cohort. (a) The ssGSEA scores from all screened genes between the two subgroups in the TCGA-PRAD cohort. (b) The ssGSEA scores from the two subgroups based on C1-upregulated genes in the TCGA-PRAD cohort. (c) The ssGSEA scores from the two subgroups based on C2-upregulated genes in the TCGA-PRAD cohort. Supplementary Figure 2: the ssGSEA scores based on C1- and C2-upregulated genes. (a) The ssGSEA scores for C1-upregulated genes between C1 and C2 subgroups were applied to total tumor entities in the TGCA cohort. (b) The ssGSEA scores based on C2-upregulated genes between the C1 and C2 subgroups applied to total tumor entities in the TCGA cohort. [file 1466255.f1.docx]
